# Variation in spawning time promotes genetic variability in population responses to environmental change in a marine fish

**DOI:** 10.1093/conphys/cov027

**Published:** 2015-07-02

**Authors:** Rebekah A Oomen, Jeffrey A Hutchings

**Affiliations:** af1 Department of Biology, Dalhousie University, Halifax, Nova Scotia, Canada B3H 4R2; af2 Centre for Ecological and Evolutionary Synthesis (CEES), Department of Biosciences, University of Oslo, Oslo 0371, Norway; af3 Department of Natural Sciences, University of Agder, Kristiansand 4630, Norway

**Keywords:** Atlantic cod, climate change, common-garden experiment, *Gadus morhua*, genotype-by-environment interaction, thermal adaptation

## Abstract

The level of phenotypic plasticity displayed within a population (i.e. the slope of the reaction norm) reflects the short-term response of a population to environmental change, while variation in reaction norm slopes among populations reflects spatial variation in these responses. Thus far, studies of thermal reaction norm variation have focused on geographically driven adaptation among different latitudes, altitudes or habitats. Yet, thermal variability is a function of both space and time. For organisms that reproduce at different times of year, such variation has the potential to promote adaptive variability in thermal responses for critical early life stages. Using common-garden experiments, we examined the spatial scale of genetic variation in thermal plasticity for early life-history traits among five populations of endangered Atlantic cod (*Gadus morhua*) that spawn at different times of year. Patterns of plasticity for larval growth and survival suggest that population responses to climate change will differ substantially, with increasing water temperatures posing a considerably greater threat to autumn-spawning cod than to those that spawn in winter or spring. Adaptation to seasonal cooling or warming experienced during the larval stage is suggested as a possible cause. Furthermore, populations that experience relatively cold temperatures during early life might be more sensitive to changes in temperature. Substantial divergence in adaptive traits was evident at a smaller spatial scale than has previously been shown for a marine fish with no apparent physical barriers to gene flow (∼200 km). Our findings highlight the need to consider the impact of intraspecific variation in reproductive timing on thermal adaptation when forecasting the effects of climate change on animal populations.

## Teaser

Common-garden experiments suggest that the response of Atlantic cod larvae to temperature differs among populations that spawn at different times of year. Populations appear to be adapted to the temperatures experienced during the larval stage at a small spatial scale, despite a lack of physical barriers to gene flow.

## Introduction

In the face of environmental disturbance, the future of a species depends on the extent to which populations respond differently to changes in their environment and the spatial correspondence between the scale of disturbance and the scale of adaptation (Hutchings *et al.*, 2007). Phenotypic plasticity is a primary mechanism by which populations might respond to environmental change in both the short and long term, by serving as a buffer against environmental variability (Canale and Henry, 2010; Nicotra *et al.*, 2010) and facilitating adaptation to new environments (Lande, 2009; Chevin *et al.*, 2010). Genetic variation in reaction norms (the range of phenotypes expressed by a genotype along an environmental gradient; Woltereck, 1909; Schmalhausen, 1949) suggests that plasticity can evolve in response to local environmental regimes (e.g. Liefting *et al.*, 2009; McCairns and Bernatchez, 2010; Baumann and Conover, 2011; De Block *et al.*, 2013). Such population variation in plasticity represents differences in the ways populations are likely to respond to directional environmental change, such as the forecasted increase in temperature due to global climate change. Understanding the mechanisms responsible for shaping population variation in responses and the spatial scales at which adaptive differences in plasticity occur is critical for predicting the persistence of a species in the face of climate change and managing populations effectively to mitigate the potential for population collapse and biodiversity loss.

Intraspecific variation in thermal reaction norms has been documented for a variety of taxa, including insects (e.g. Van Asch et al., 2007; Winterhalter and Mousseau, 2007; Liefting and Ellers, 2008), reptiles (e.g. Sinervo and Adolph, 1994; Bronikowski, 2000), amphibians (e.g. Ficetola and De Bernardi, 2005; Richter-Boix *et al.*, 2010) and fishes (e.g. Conover and Present, 1990; Yamahira *et al.*, 2007; Jensen *et al.*, 2008). Such variation is usually quantified at broad spatial scales and is quite possibly a result of adaptation to seasonality or temperature along a latitudinal cline. Population differences in thermal responses have also been reported at very small spatial scales when thermal regimes differ between nearby habitats [e.g. 5–20 km in the garter snake, *Thamnophis elegans* (Bronikowski, 2000); <1 km in the soil arthropod, *Orchesella cincta* (Liefting and Ellers, 2008)] or altitudes [e.g. 50–60 km in the frog, *Rana latastei* (Ficetola and De Bernardi, 2005)].

Environments can vary both spatially and temporally. For traits related to reproduction or early life stages, intraspecific variation in the timing of reproduction can theoretically promote adaptive variability independent of geographical variation due to temporal variability in selective pressures. Studies of adaptive divergence among groups of individuals that reproduce at different times of year (i.e. isolation-/adaptation- by-time) are seemingly rare, despite variation in reproductive timing existing in a wide variety of taxa (reviewed by Hendry and Day, 2005). Richter-Boix *et al.* (2013) demonstrated a correlation between larval life-history traits and reproductive timing among moor frog (*Rana arvalis*) populations that each have an explosive breeding period of a few days within a 22 day span. Lower growth rate and a longer larval period were associated with early breeding and wetlands with warmer water temperatures, despite high gene flow throughout the study area. On a broader temporal scale, Fraser *et al.* (2011) suggested the potential for adaptive divergence between spring, summer and winter breeding runs within rivers in some salmonids (e.g. Waples *et al.*, 2004), although this has not been explicitly tested. Given that thermal regimes can vary drastically through the year in temperate climates, variation in reproductive timing might promote cryptic genetic variation in thermal responses within species.

We examined genetic variation in early life-history trait plasticity in Atlantic cod (*Gadus morhua*; hereafter, cod), a demersal marine fish of widespread ecological and socioeconomic importance that is threatened over much of its range by the compound effects of climate change and overfishing (O'Brien *et al.*, 2000; Committee On the Status of Endangered Wildlife In Canada, 2010; Hutchings and Rangeley, 2011). The collapse of Canadian cod stocks in the early 1990s was biologically, socially and economically devastating (Templeman, 2010; Hutchings and Rangeley, 2011). Despite a moratorium on fishing since 1992, most stocks have shown little or no recovery (Hutchings, 2000; Hutchings and Rangeley, 2011). However, variable rates of recovery among stocks underscore the need to understand the underlying genetic differences among stocks for traits that are likely to be influencing recovery.

Cod inhabit coastal waters throughout the North Atlantic characterized by a variety of thermal regimes that promote localized thermal adaptation (Hutchings *et al*., 2007; Bradbury *et al.*, 2010, 2013). In addition, different groups of cod spawn at different times of year (e.g. Lett, 1980; Brander and Hurley, 1992; Myers *et al.*, 1993). Some management units, such as the Southern designatable unit (DU) on the Southwestern Scotian Shelf in Canada (Committee On the Status of Endangered Wildlife In Canada, 2010), contain multiple spawning components that collectively spawn through most of the year (from September to June; Brander and Hurley, 1992). The temporally stable genetic structure among local spawning groups (reviewed by Ruzzante *et al.*, 1999) and persistent differences in spawning times among populations kept in a common environment over multiple years (Otterå *et al*., 2006) suggest that spawning time is, in part, heritable in cod, as in other fishes (e.g. Siitonen and Gall, 1989; Danzmann *et al.*, 1994; Sakamoto *et al.*, 1999; Rogers *et al.*, 2006). Therefore, variation in spawning times is expected to manifest in adaptive genetic differences at small spatial scales. Indeed, Marcil *et al.* (2006) found differences in body shape plasticity between neighbouring spawning components of the Scotian Shelf that experience peak spawning 1 month apart. However, the adaptive significance of these differences is not known.

We constructed thermal reaction norms for two adaptive traits: larval growth (whereby faster growth increases fitness by reducing the duration of the vulnerable pelagic larval phase; Anderson, 1988) and survival. We compared these responses among groups of cod that spawn at different times of year (hereafter, ‘spawning groups’ and ‘populations’ are used interchangeably). Our objectives were to investigate the role of variation in spawning time in promoting adaptive divergence in thermal plasticity in cod and to determine whether genetic variation in plasticity for adaptive traits exists among spawning groups at a smaller spatial scale than that of the current management units. We discuss the short- and long-term implications of our findings in light of predicted changes in climate.

## Materials and methods

### Study populations

**Figure 1:**
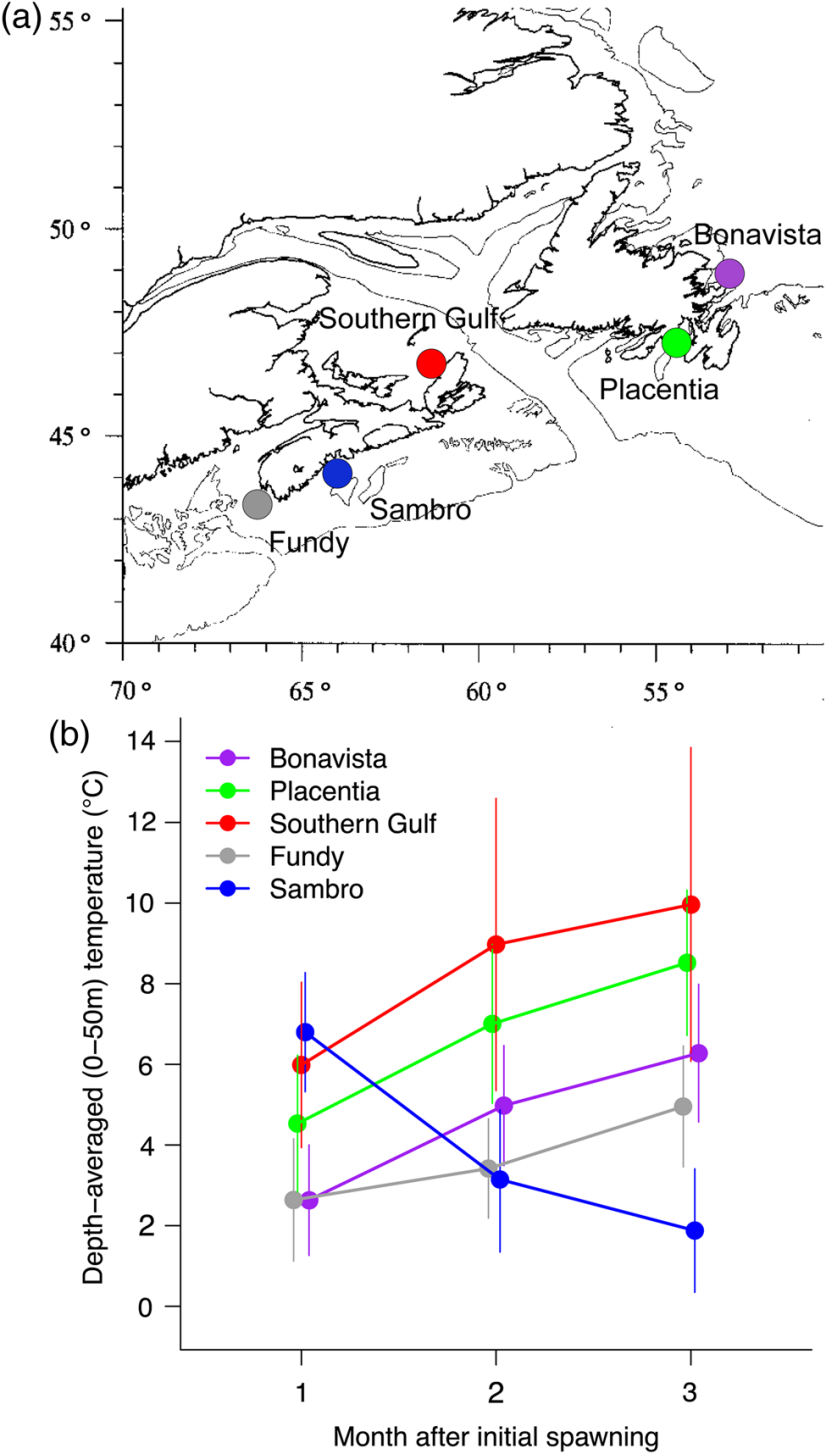
(**a**) Sampling locations of spawning adults for study populations of Atlantic cod (*Gadus morhua*). (**b**) Depth-averaged (0–50 m) water temperatures (in degrees Celsius ± 1 SD) for the first 3 months after the initial peak spawning months [May (Bonavista, Placentia and Southern Gulf), February (Fundy) and November (Sambro)]. Mean temperatures were estimated by using all available data from 1914 to 2009 in the Bedford Institute of Oceanography's Hydrographic Climate Database (http://www.bio.gc.ca/science/data-donnees/base/climate-climat-eng.php).

Six common-garden experiments were conducted on the following five populations of cod that were collected between 218 and 1140 km apart (Fig. 1a): (i) Bay of Fundy [Northwest Atlantic Fisheries Organization (NAFO) division 4X; Southern DU]; (ii) Southwestern Scotian Shelf near Sambro, Nova Scotia (NAFO division 4X; Southern DU); (iii) Southern Gulf of St Lawrence (NAFO division 4T; Laurentian South DU); (iv) Bonavista Bay, Newfoundland (NAFO division 3L; Newfoundland and Labrador DU); and (v) Placentia Bay, Newfoundland (NAFO division 3Ps; Laurentian North DU; Committee On the Status of Endangered Wildlife In Canada, 2010). Cod from these areas will be referred to as Fundy, Sambro, Southern Gulf, Bonavista and Placentia, respectively.

Fundy and Sambro cod are two of what are thought to be multiple spawning components in the Southwestern Scotian Shelf, based in part on spatial differences in spawning times. Peak spawning of Fundy cod occurs in February (Hutchings *et al.*, 2007), while Sambro cod have a peak spawning period from November to December (Brander and Hurley, 1992; Hutchings *et al.*, 1999). The remaining populations experience peak spawning from May to June (Lett, 1980; Myers *et al.*, 1993). Due to differences in spawning times and locations, populations experience different temperature regimes during early life (Fig. 1b; see Supplementary Fig. 1 for year-round temperature profiles). Fundy and Sambro larvae experience relatively cold temperatures, with more stable temperatures for Fundy compared with Sambro, while Southern Gulf, Bonavista and Placentia larvae experience relatively warm and variable temperatures.

### Common-garden experiments

Common-garden experiments on Fundy, Southern Gulf, Bonavista and Placentia cod were conducted by Hutchings *et al.* (2007) from 2002 to 2003. We performed additional experiments on Sambro and Southern Gulf cod from 2011 to 2012, using similar protocols. Briefly, the methods are as follows.

Between 34 and 73 wild-caught adults from each population were obtained during (Bonavista only) or immediately prior to their respective breeding seasons, i.e. in June 2003 (Bonavista), April 2002 (Placentia), May 2003 and 2011 (Southern Gulf), January 2002 (Fundy) and November 2011 (Sambro). Adult cod spawned undisturbed either in the 684 m^3^ Pool Tank in the Aquatron Laboratory at Dalhousie University (Fundy, Sambro and Southern Gulf) or at the Oceans Sciences Centre at Memorial University of Newfoundland (Bonavista and Placentia); all cod were held at ∼8°C.

Common-garden experiments took place at Dalhousie University (Sambro and Southern Gulf 2011) or at the Ocean Sciences Centre (Fundy, Southern Gulf 2003, Bonavista and Placentia), to which fertilized eggs from Dalhousie University were transported if necessary. Fertilized eggs were collected 2–4 weeks after they were first observed in mesh collectors positioned at the surface outflows of the tanks. Eggs were incubated at 7°C until hatch, at which time larvae were transferred to 20 l (Dalhousie) or 30 l tanks (Memorial). Larvae were reared at two temperatures (7°C ± 1 and 11°C ± 1°C) with three (Sambro only) or four replicate tanks per treatment and 1200 larvae per replicate. On the day of transfer, all tanks were set to 7°C. The following day, the water in the high-temperature treatments was gradually changed to 11°C over the course of 12 h. Larvae were fed rotifers in excess (4500 prey/litre, corresponding to the high-food treatment of Hutchings *et al*. 2007), three times per day (at ∼09,00, 13.00 and 17.00 h). Larvae were fed *Isochrysis*-enriched rotifers from day 1 to 10, Ori-Green (Skretting)-enriched rotifers from day 11 to 31, a 1:1 mixture of rotifers and *Artemia* from day 32 to 39 and *Artemia* only from day 40 to 43. Larvae were reared under a light intensity of 2000 lux, and water temperatures were monitored daily. Standard length at hatch was measured for 40–80 (mean = 65) randomly sampled larvae per population using AxioVision image analysis software (Zeiss). Length at 29 days post-hatch was measured for 10 randomly sampled larvae per replicate (except for Fundy and Bonavista, for which five and six larvae were measured per replicate, respectively) and used as a proxy for growth following Hutchings *et al*. (2007). Survival was quantified as the mean number of larvae alive in each tank on day 43 relative to day 0 and was not corrected for sampling mortality.

### Evaluation of the number of families

To evaluate the assumption that a substantial number of families from each population were represented in the experiment, adults (post-spawning) and larvae were genotyped at five to seven microsatellite loci, according to the methods described by Hardie *et al.* (2006). Genotypes were analysed using PAPA v.2.0 (Duchesne *et al.*, 2002) or COLONY (Jones and Wang, 2010) to assign parentage (see Hutchings *et al*., 2007; and Supplementary data for further detail). Based on these analyses, the number of families represented in the experiment was at least 21, 29, 31, 15, 71 and 44 for Fundy, Sambro, Southern Gulf (in 2003), Southern Gulf (in 2011), Bonavista and Placentia, respectively. In general, similar minimal numbers of families were identified in samples from the beginning and end of the experiments (excepting Fundy, but note differences in sample sizes between time points) and genetic differentiation between time points within experiments was low (Supplementary data).

### Reaction norm analyses

All statistical analyses were performed in R (R Development Core Team, 2012). We performed a one-way analysis of variance (ANOVA) on length at hatch to determine whether it differed among populations and tested for linear relationships between length at hatch and reaction norm slopes. Using a linear mixed-effects model, we constructed reaction norms for larval growth for each population. Population, temperature and their interaction were used as fixed effects and tank as a random effect nested within temperature. To facilitate visual comparison of reaction norm slopes, mean larval length at each temperature was plotted relative to the lowest mean length observed in each population.

Assuming that reaction norm variation is greater among families than within them, there is the potential for pseudoreplication to increase the power of the test artificially for population differences in growth. We invalidated this assumption for Sambro (the only experiment for which family data were available; for details see Supplementary data). We do not know if the same is true for the remaining populations. However, the minimal number of families present at the end of the Bonavista and Placentia experiments (Supplementary data) exceeds the sample size for the growth analysis (*n* = 40); therefore, the probability of resampling a particular family is low. Thus, the potential impact of pseudoreplication on the test for population differences in growth is likely to be minimal.

To resolve whether variation in reaction norms could be attributed to the fact that experiments were conducted at different times and locations with slightly different protocols, we compared reaction norms for two experiments involving Southern Gulf cod that were carried out in 2003 and 2011. A two-way ANOVA revealed that, although the elevations (i.e. mean trait values) of the reaction norms differed for growth (*F* = 5.94; *P*_1,82_ = 0.017; Supplementary data) and survival (*P* < 0.001; Supplementary data), the slopes did not (*F* = 0.957; *P*_1,82_ = 0.331 and *P* = 0.932 for growth and survival, respectively). Thus, comparison of reaction norm slopes among these experiments is unlikely to be confounded by temporal or experimental variation, although the same cannot be said for reaction norm elevations. Given that the mean trait values differed between the Southern Gulf experiments, the data could not be combined. Of the two Southern Gulf experiments, we retained only the 2003 experiment for further analyses due to higher mortality in the 2011 experiment resulting in the early termination of some tanks. This elevated mortality was unrelated to temperature (*P* = 0.328; Supplementary data).

To evaluate whether variation in density among tanks due to differential survival may have contributed to tank effects observed in the growth model, we examined whether there was a relationship between the magnitude and direction of random effects and tank density. We used survival on day 43 as a proxy for density. A plot of random effect size against survival showed no association (Supplementary Fig. 2); therefore, differential tank density was not considered to be responsible for variation in growth among tanks.

We constructed survival reaction norms using back-transformed model estimates from a generalized linear model with a quasi-binomial distribution and logit link. Population, temperature and their interaction were the fixed effects. Mean survival at each temperature was then plotted relative to the highest mean survival observed in each population. To test for a significant genotype × environment interaction, the identity link was used instead of the logit link so that the reaction norm intercepts did not influence the test.

## Results

### Reaction norm variation

Larval length at hatch differed among populations (*F*_5_ = 117.13; *P* < 0.001), with Fundy and Sambro larvae generally being larger than the remaining populations (Supplementary data). However, there was no relationship between length at hatch and growth (*F* = 0.68; *P*_1,3_ = 0.47) or survival (*F* = 0.11; *P*_1,3_ = 0.76) responses.

**Figure 2:**
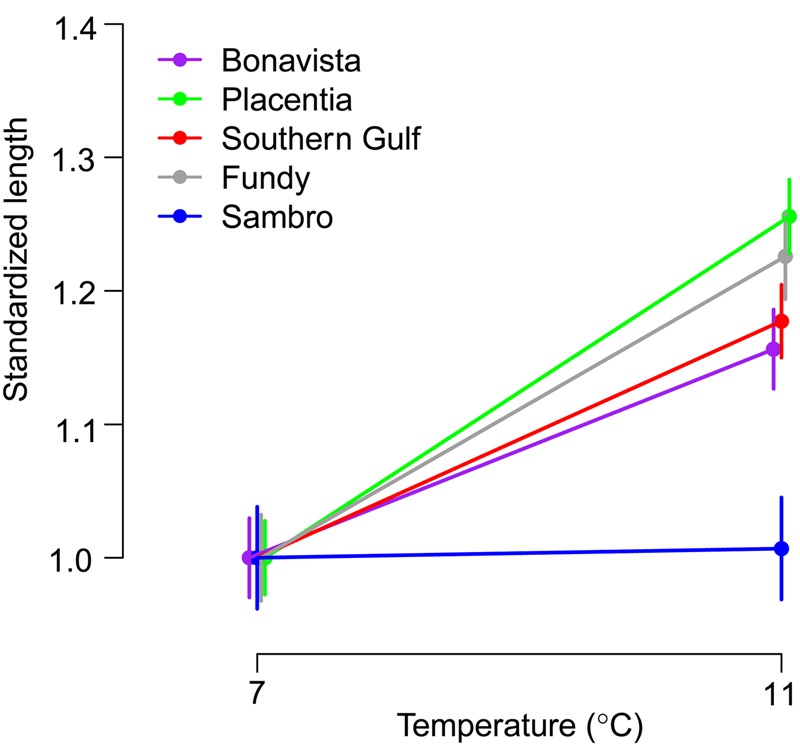
Thermal reaction norms for larval cod growth (±1 SEM), standardized relative to the lowest mean length observed in each population.

We found substantial population variation in thermal responses for growth (Fig. 2), manifested by a significant population × temperature interaction (*F* = 4.78; *P*_4,261_ = 0.001; Table 1). Plasticity for growth in response to temperature was evident in all populations except for Sambro and was such that larvae grew faster with the high-temperature treatment (Table 2). In contrast, growth of Sambro larvae did not differ between temperature treatments (*t* = 0.13, *P* = 0.450). After correcting for multiple comparisons, differences in reaction norm slopes were significant or marginally significant between Sambro and all other populations except Bonavista, for which the difference was significant before the correction (Table 3). Among the populations that exhibited plasticity, the magnitudes of the responses were similar. The uncorrected results suggest that the slope of the response of Placentia larvae was steeper than that of Bonavista (*t* = 1.79, *P* = 0.043) and marginally steeper than that of Southern Gulf (*t* = −1.33, *P* = 0.097; Table 3). However, these differences were not significant after correcting for multiple comparisons.

**Table 1:** Effects of population and temperature on larval cod growth

Model term	d.f.	Sum of squares	Mean of squares	*F*	*P*-value
Population	4	36.74	9.19	28.38	<0.001*
Temperature	1	25.92	25.92	80.07	<0.001*
Population × temperature	4	6.19	1.55	4.78	0.001*
Model term		Variance	SD		
Tank		0.15	0.39		
Residual		0.32	0.57		

Asterisk denotes significance at α = 0.05.

**Table 2:** Effect of temperature on larval growth for five cod populations, where the estimate represents the change in growth from 7 to 11°C

Population	Estimate	SEM	*t*	*P*-value
Bonavista	1.19	0.32	3.72	<0.001*
Placentia	1.97	0.30	6.54	<0.001*
Southern Gulf	1.38	0.33	4.25	<0.001*
Fundy	1.62	0.33	4.95	<0.001*
Sambro	0.04	0.35	0.13	0.450

Asterisk denotes significance at α = 0.05 after Bonferroni correction.

**Table 3:** Pairwise population contrasts of the effect of temperature on larval cod growth

	Bonavista	Placentia	Southern Gulf	Fundy	Sambro
Bonavista	—	0.79 (±0.44)	0.20 (±0.46)	0.44 (±0.46)	−1.14 (±0.47)
Placentia	0.043††	—	−0.59 (±0.44)	−0.35 (±0.45)	−1.93 (±0.46)
Southern Gulf	0.336	0.097†	—	0.24 (±0.46)	−1.34 (±0.48)
Fundy	0.177	0.219	0.308	—	−1.58 (±0.48)
Sambro	0.012††	<0.001**	0.004*	0.002**	—

Estimates (±SEM) are given above the diagonal and *P*-values below. The point of contrast for the estimates is the row header. Symbols denote significance at the following levels of α: *0.10 and **0.05 (with Bonferroni correction), †0.10 and ††0.05 (without Bonferroni correction). A Bonferroni correction for all contrasts of interest (*n* = 15) changes the critical *P*-values to 0.007 (α = 0.10) and 0.003 (α = 0.05).

**Figure 3:**
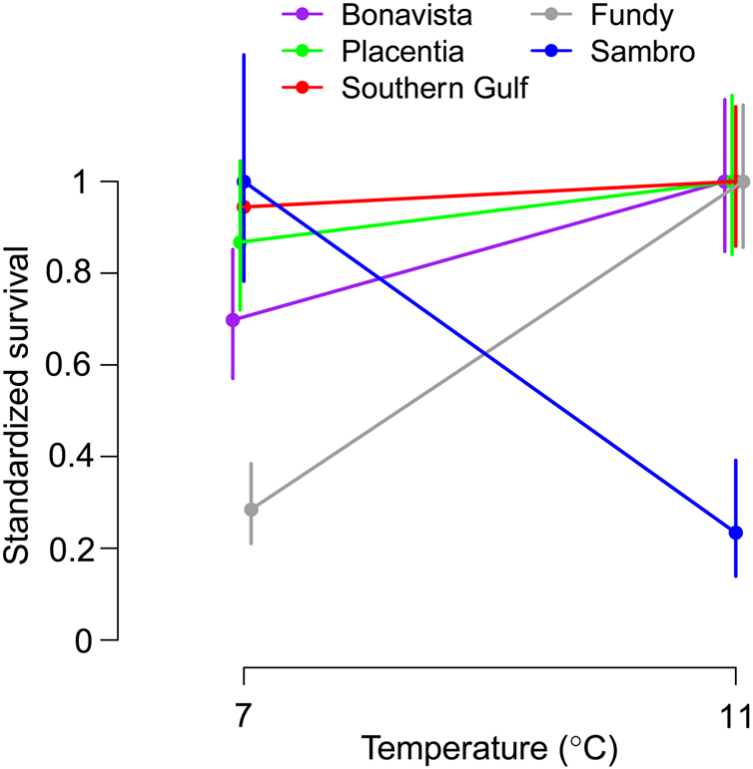
Thermal reaction norms for larval cod survival (±1 SEM), standardized relative to the highest mean survival observed in each population.

Significant variation in survival reaction norm slopes was observed between populations (*P* < 0.001; Fig. 3 and Table 4). Fundy larvae exhibited a high degree of plasticity, with a significant positive relationship between survival and temperature (*P* < 0.001; Table 5) and 2.5 times greater survival in the high-temperature treatment. The opposite response was observed in Sambro larvae, with survival for the low-temperature treatment being more than three times greater than survival for the high-temperature treatment, although this effect was not significant after correcting for multiple comparisons (*P* = 0.009). Several populations (Southern Gulf, Bonavista and Placentia) exhibited no plasticity. The slopes of these populations were significantly (Placentia and Southern Gulf; *P* = 0.031 for both) or marginally significantly (Bonavista; *P* = 0.108) different from that of Fundy and significantly (Bonavista and Placentia; *P* = 0.010 and *P* = 0.044, respectively) or marginally significantly (Southern Gulf; *P* = 0.092) different from Sambro (Table 6). However, none of these differences was significant after a Bonferroni correction. Only the responses of Fundy and Sambro larvae were significantly different after correcting for multiple comparisons (*P* < 0.001).

**Table 4:** Deviance table of the effects of population and temperature on larval cod survival

Model term	d.f.	Deviance	Residual d.f.	Residual deviance	*P*-value
Null			37	608.96	
Population	4	176.37	33	432.59	0.010*
Temperature	1	15.31	32	417.28	0.276
Population × temperature	4	190.38	28	226.90	<0.001*

The *P*-values were obtained from χ^2^ tests that were used to determine if the model fit improved significantly by sequentially adding population, temperature and their interaction to the null model. Asterisk denotes significance at α = 0.05.

**Table 5:** Effect of temperature on larval survival for five cod populations, where the estimate represents the change in survival from 7 to 11°C

Population	Estimate	SEM	*t*	*P*-value
Bonavista	0.01	0.01	1.39	0.175
Placentia	0.01	0.01	0.56	0.582
Southern Gulf	0.00	0.01	0.27	0.792
Fundy	0.04	0.01	3.96	<0.001*
Sambro	−0.02	0.01	−2.71	0.011†

Symbols denote significance at α = 0.05 with (*) and without (†) Bonferroni correction.

**Table 6:** Pairwise population contrasts of the effect of temperature on larval cod survival

	Bonavista	Placentia	Southern Gulf	Fundy	Sambro
Bonavista	—	−0.96 (±1.64)	−1.25 (±1.76)	2.60 (±1.57)	−4.05 (±1.46)
Placentia	0.563	—	−0.29 (±1.76)	3.56 (±1.57)	−3.10 (±1.47)
Southern Gulf	0.483	0.870	—	3.85 (±1.70)	−2.80 (±1.61)
Fundy	0.108	0.031††	0.031††	—	−6.65 (±1.39)
Sambro	0.010††	0.044††	0.092†	<0.001**	—

Model estimates (±SEM) are given above the diagonal and *P*-values below. The point of contrast for the estimates is the row header. Symbols denote significance at the following levels of α: *0.10 and **0.05 (with Bonferroni correction), †0.10 and ††0.05 (without Bonferroni correction). A Bonferroni correction for all contrasts of interest (*n* = 15) changes the critical *P*-values to 0.007 (α = 0.10) and 0.003 (α = 0.05).

## Discussion

### Plasticity in growth and survival

We found variation in thermal reaction norms for larval growth and survival among five putative populations of Atlantic cod in the Northwest Atlantic. Four populations (Fundy, Southern Gulf, Bonavista and Placentia) presented highly plastic growth responses to temperature, growing faster in warmer water, whereas there was no evidence of plasticity for growth in Sambro cod. The magnitude of change might also differ slightly among the plastic growth responses, with Placentia exhibiting the greatest response, but further study will be needed to confirm the significance of this subtle variation. An even greater variety of responses to temperature was observed for survival. Fundy cod experienced much higher survival in warmer water, while the opposite was true for Sambro cod. Survival of Southern Gulf, Bonavista and Placentia larvae was not significantly influenced by temperature.

We interpret these differences in plasticity for larval growth and survival to be largely of genetic rather than maternal origin. This is supported by a lack of relationship between variation in size at hatch (influenced by egg size, the main cause of maternal effects in fishes, Marshall, 2008) and growth or survival, although epigenetic maternal effects cannot be ruled out (e.g. Miller *et al.*, 2012). In a rare example of transgenerational plasticity to temperature in vertebrates, Salinas and Munch (2012) observed optimal growth in larval sheepshead minnows (*Cyprinodon variegatus*) at temperatures recently experienced by the parents. In contrast, most populations in our study (excepting Sambro) grew more slowly at the colder temperature that was most similar to that at which the parents were held during spawning (8°C) and the temperatures experienced prior to collection (1–4°C; Supplementary Fig. 1). Therefore, transgenerational plasticity does not seem to explain the variation in thermal responses we observed, although it might play an important role in population responses to climate change.

Other potential causes of reaction norm variation in these experiments include variation in larval density among tanks and the fact that experiments were carried out at different times and locations. Stocking densities of 50–300 larvae/litre have been shown to have no effect on survival or growth of cod larvae as long as food is not limiting (Baskerville-Bridges and Kling, 2000), as was the case in our experiment. We also showed that random effects in the growth model were not related to tank density. A lack of association between low survival and high growth further supports our conclusion that variation in density is unlikely to be responsible for the patterns of phenotypic variation observed.

The fact that the common-garden experiments were conducted at different times and locations with slight variation in protocol might have influenced the mean trait values of the reaction norms. However, the reaction norm slopes are unlikely to be significantly affected, as demonstrated by a lack of difference in growth plasticity between the two Southern Gulf experiments. Hutchings *et al.* (2007) also found no differences in length within treatments between two experiments on Fundy cod that were conducted 1 year apart using the same protocol. Additional replicate experiments are needed to assess the temporal stability of reaction norms and robustness of the common-garden experiments. Perhaps with changing ocean temperatures and sufficient time scales for evolution in plasticity to occur, temporal variation in thermal reaction norm slopes could become apparent (Crozier and Hutchings, 2014).

High mortality is typical of cod larvae in the wild (Sundby *et al.*, 1989; Houde and Zastrow, 1993) and in the laboratory (Gamble and Houde, 1984; Otterlei *et al.*, 1999; Steinarsson and Björnsson, 1999). In our study, survival (uncorrected for sampling mortality) ranged from 0.7 to 5.5% and no population consistently exhibited the highest or lowest survival. If size-selective mortality was responsible for the differences in length observed between groups, we would expect to see a consistent correlation between length and survival among populations, which was not the case. The cause of the exceptionally low survival in the 2011 Southern Gulf experiment is unknown, although survival was not associated with temperature in 2003 or 2011. Interestingly, growth reaction norms did not differ between the two Southern Gulf experiments despite different survival rates. These lines of evidence suggest that variation in survival does not explain the observed differences in growth between temperatures or populations.

### Thermal adaptation in plasticity

Three groups are evident based on the types of responses they exhibit to increased temperature: faster growth and higher survival (Fundy); faster growth and similar survival (Southern Gulf, Bonavista and Placentia); and similar growth and lower survival (Sambro). These groups can also be characterized as winter-spawning, spring-spawning and autumn-spawning populations, respectively, each of which experiences a unique pattern of thermal variability in early life (Fig. 1b). While geography and reproductive timing are somewhat interdependent (e.g. the three more northerly populations all spawn in the spring), the two populations with the greatest proximity to one another (Fundy and Sambro) spawn at different times and exhibit the most divergent thermal responses, while the next most proximal populations (Bonavista and Placentia) spawn at the same time and show similar responses. Therefore, variation in reproductive timing rather than geography corresponds better with the variation in thermal responses observed.

The association between thermal reaction norms and the timing of the spawning season raises the question as to the specific mechanism responsible for shaping these diverse responses. Fundy and Sambro cod experience the coldest temperatures overall, yet their reaction norms are the most divergent. Therefore, the average temperature experienced during the larval stage is insufficient to explain the observed reaction norm variation. An association between plasticity and thermal variability is not evident either; Fundy and Sambro both experience relatively low levels of thermal variability compared with the remaining populations due to intense vertical mixing that homogenizes the water column (Garrett *et al.*, 1978; Drinkwater and Gilbert, 2004). The northerly spring-spawning populations could have evolved an intrinsically faster growth rate to compensate for a shorter growing season, as has been found in other fishes (e.g. Conover and Present, 1990; Schultz *et al.*, 1996; Conover *et al.*, 1997), with limited evidence in Northwest Atlantic cod (Hunt von Herbing *et al.*, 1996; Purchase and Brown, 2000). However, growing season does not explain the marked difference in growth response between Fundy and Sambro cod, which both experience the coldest, though unlikely to be growth-limiting, temperatures of the year during the first few months after spawning. Rather, it is the magnitude and direction of the seasonal change in post-spawning temperature that seem to provide the best explanation for the observed reaction norm variability, such that winter- and spring-spawning populations appear to be adapted to increasing temperatures and those populations that experience colder temperatures overall are more sensitive to changes in temperature.

Previous studies have found that cold-water populations experience increasing survival with temperature (Planque and Frédou, 1999; Worm and Myers, 2003; Ottersen *et al.*, 2006). However, Sambro larvae exhibited decreasing survival with temperature, despite experiencing relatively cold temperatures in the wild. What distinguishes the thermal regime typical for Sambro larvae is the decrease in temperature during the larval stage. As a consequence, Sambro cod may not have experienced the selective pressures necessary to shape an adaptive norm of reaction for growth to higher temperatures. This lack of plasticity is probably maladaptive at high temperatures, especially considering the corresponding decrease in survival. The thermal response of Sambro larvae might be the result of a trade-off between having high performance in the natural environment at the expense of low performance in others (i.e. specialist–generalist trade-off), such as observed in Atlantic salmon (*Salmo salar*; Rungruangsak-Torrissen *et al.*, 1998), or selection for energy savings at low temperatures (Pörtner *et al.*, 2008). Further information about the physiological mechanisms (e.g. gene expression) that underlie the various responses we observed could help distinguish between different trade-offs that might be responsible (Angilletta *et al.*, 2003).

### Spatial scale of genetic variation

We found genetic variation in thermal reaction norms between two cod spawning components sampled 218 km apart within the same fisheries management unit (4X). However, the geographical ranges occupied by these spawning groups are not known and may even overlap; therefore, the spatial scale of probable adaptive divergence is likely to be smaller than the distance between collection locations. This is the smallest spatial scale at which genetic variation in adaptive traits has been detected across open waters in a marine fish, that is, waters that are not physically separated by land in some manner, such as along coastal Norway (e.g. Olsen *et al.*, 2008). The finding of this fine-scale biocomplexity in a species that is widely distributed and has high potential for dispersal contradicts traditional notions of genetic homogeneity in marine systems (reviewed by Hilbish, 1996).

The degree to which the autumn- and winter-spawning components of the Scotian Shelf intermix is not known. Given the considerable differences in spawning times, and the limited migration and apparently low levels of gene flow between the winter-spawning components (Ruzzante *et al.*, 1998), differentiation of Sambro cod from winter-spawning components at neutral markers seems likely. However, the significant genetic variation in reaction norms among the remaining populations is not matched by differentiation at neutral markers (Hardie *et al.*, 2006; Hutchings *et al.*, 2007). The lack of correspondence between neutral and adaptive markers provides evidence of genetic structure resulting from selection persisting in the face of apparently high gene flow (Hutchings *et al.*, 2007).

### Implications for climate change

Our study suggests that variation in the timing of reproduction has the potential to promote genetic variability in population responses to environmental change in species with high dispersal capabilities at a very small spatial scale. Intraspecific variation in reproductive timing is common in nature and is often correlated with variation in phenotypic traits (reviewed by Hendry and Day, 2005). While variation in reproductive timing can be linked with temperature, allowing populations to track climate change by adjusting breeding times as global temperatures rise (e.g. Charmantier *et al.*, 2008), this is not always the case. In cod, there is no consistent relationship between spawning onset and water temperature in the Northwest Atlantic (Brander and Hurley, 1992; Myers *et al.*, 1993), possibly because the effect of temperature depends on local hydrography and migratory behaviour (Hutchings and Myers, 1994). Thus, it is less likely that cod populations will be able to track climate change by adjusting spawning times.

Even a small, sustained change in ocean temperature could have substantial impacts on population growth rate and recovery considering the high levels of plasticity in life-history traits in some cod populations (Drinkwater, 2005). Our findings suggest that the forecasted 2–4°C rise in ocean temperatures for the study regions (IPCC, 2007) will affect populations differently depending on spawning time (e.g. an increase in productivity for winter- and spring-spawning populations but a decline in that of autumn-spawning populations). Of course, these outcomes will depend on numerous ecosystem variables, such as changes in food availability (Stenseth and Mysterud, 2002; Pörtner and Peck, 2010) and the extent to which cod are able to alter their distribution as temperature changes, given that they are highly mobile (e.g. Neat and Righton, 2007; Freitas *et al.*, 2015). Nonetheless, the thermal responses described in the present study provide a strong empirical basis for predictions of climate change impacts on the abundance and distribution of cod. Furthermore, they highlight the need to consider ecological and behavioural factors that may influence thermal responses in addition to the geographical characteristics typically studied, such as latitude (e.g. Yamahira *et al.*, 2007; Berger *et al.*, 2013; De Block *et al.*, 2013) and altitude (e.g. McKenzie *et al.*, 2013; Vitasse *et al.*, 2013).

The long-term (i.e. evolutionary) consequences of climate change on cod populations will depend on the amount of heritable variation in reaction norms they possess. Variation in adaptive plasticity at the population level increases the likelihood of at least one population having a response that is beneficial in the new environment. The variety of responses observed in the present study alone would suggest that at least one cod population would be well suited to any (small) directional change in temperature, although such a change would most probably result in a loss of intraspecific biodiversity overall through declines (or in the worst case, extinctions) of those populations ill suited to the new environment (Bálint *et al.*, 2011; Pauls *et al.*, 2013). Selection can also act on variation contained within populations to shape a norm of reaction that is adapted to future thermal environments (Ghalambor *et al.*, 2007). Future research should seek to quantify the variation in plasticity that exists within populations (e.g. at the family level) in order to assess the adaptive potential of individual populations (Hutchings 2011).

Many species face additional natural and anthropogenic threats that might interact with climate change, such as habitat fragmentation, pathogens or overexploitation (IPCC, 2014). In harvested species, the negative impacts of climate change can be exacerbated by overexploitation (Hilborn *et al.*, 2003; Hutchings and Reynolds, 2004; Mora *et al.*, 2007). Prevention of further loss of biodiversity and promotion of the recovery of depleted populations will require management strategies that consider both ecological and evolutionary responses of species to their ever-changing environments that are based on appropriate spatial scales (Conover *et al.*, 2006; Hutchings *et al.*, 2007; Olsen *et al.*, 2008). This approach will help to ensure that the adaptive diversity contained in unique populations is preserved and that species have the best genetic tools to cope with their changing environment.

## Supplementary material

Supplementary material is available at *Conservation Physiology* online.

## Funding

This work was supported by the Natural Sciences and Engineering Research Council through Strategic and Discovery Grants to J.A.H. and a Canada Graduate Scholarship to R.A.O.; Loblaw Companies Ltd; and the Canadian Wildlife Federation.

## Supplementary Material

Supplementary Data
