# Supplementary material for: Variation in spawning time promotes genetic variability in population responses to environmental change in a marine fish
Source: Conserv Physiol. 2015 Jul 2;3(1):cov027. doi: 10.1093/conphys/cov027 (PMC4778481; doi:10.1093/conphys/cov027)
Supplement: Supplementary Data [file cov027supp.zip › cov027supp_fig1.pdf]

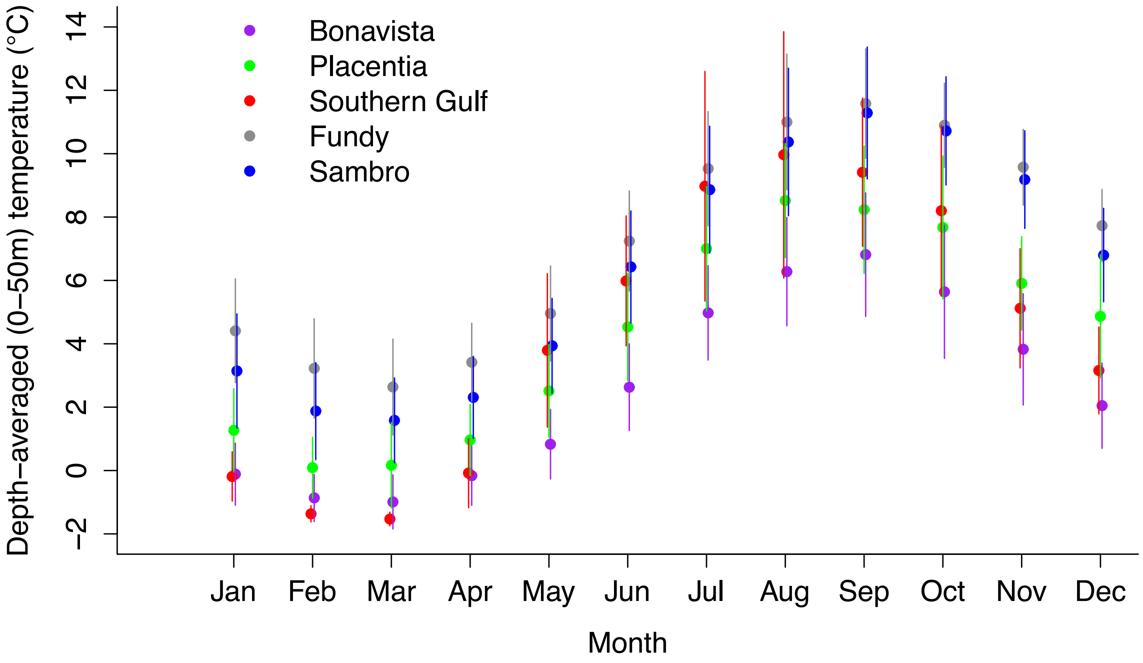

Supplementary Figure 1: Monthly depth-averaged (0-50 m) water temperatures (°C ±1 SD). Mean temperatures were estimated by using all available data from 1914-2009 in the Bedford Institute of Oceanography's Hydrographic Climate Database (http://www.bio.gc.ca/science/data-donnees/base/climate-climat-eng.php).
