# Supplementary material for: Variation in spawning time promotes genetic variability in population responses to environmental change in a marine fish
Source: Conserv Physiol. 2015 Jul 2;3(1):cov027. doi: 10.1093/conphys/cov027 (PMC4778481; doi:10.1093/conphys/cov027)
Supplement: Supplementary Data [file cov027supp.zip › cov027supp_table1.pdf]

Supplementary Table 1: Larval cod survival statistics and the minimum numbers of families identified at the beginning and end of the common-garden experiments. The numbers of individuals for which parentage was assigned are given in parentheses. The level of genetic differentiation between initial and final samples was evaluated using F_{ST} in Arlequin v.3.5 (Excoffier *et al.*, 2005).

Population	Spawning season	Initial # families (n)	Temperature (°C)	# replicates	Mortality (%)	Mean survival	Total survival	Final # families (n)	F_{ST}	*P*
Bonavista	Spring	na	7	4	96.60	61.50	246	46 (115)	na	na
			11	4	95.12	42.25	169	45 (145)	na	na
Placentia	Spring	66 (194)	7	4	96.12	48.50	194	62 (na)	0.007	<0.001
			11	4	95.52	56.25	225	47 (132)	0.005	<0.001
Southern Gulf	Spring	48 (415)	7	4	94.66	67.75	271	25 (154)	0.012	<0.001
			11	4	94.34	72.00	288	29 (166)	0.007	<0.001
Fundy	Winter	38 (584)	7	4	98.44	19.00	76	3 (3)	-0.043	1.000
			11	4	94.53	69.50	278	19 (135)	0.004	<0.001
Sambro	Autumn	22 (120)	7	3	96.93	38.00	114	11 (30)	-0.003	0.634
			11	3	99.28	8.67	26	12 (29)	-0.004	0.803
