# Supplementary material for: Variation in spawning time promotes genetic variability in population responses to environmental change in a marine fish
Source: Conserv Physiol. 2015 Jul 2;3(1):cov027. doi: 10.1093/conphys/cov027 (PMC4778481; doi:10.1093/conphys/cov027)
Supplement: Supplementary Data [file cov027supp.zip › cov027supp_table2.pdf]

Supplementary Table 2: Effects of year and temperature on larval cod growth for Southern Gulf experiments conducted in 2003 and 2011.

Model term	df	Sum of squares	Mean of squares	F	*P*	
year	1	2.42	2.42	5.94	0.017	**
temperature	1	10.91	10.91	26.74	<0.001	**
year × temperature	1	0.39	0.39	0.96	0.331	
Model term		Variance	Standard deviation			
tank		0.22	0.47			
residual		0.41	0.64			

Asterisks denote significance at the following levels of α: * = 0.10, ** = 0.05.
