# Supplementary material for: Variation in spawning time promotes genetic variability in population responses to environmental change in a marine fish
Source: Conserv Physiol. 2015 Jul 2;3(1):cov027. doi: 10.1093/conphys/cov027 (PMC4778481; doi:10.1093/conphys/cov027)
Supplement: Supplementary Data [file cov027supp.zip › cov027supp_table3.pdf]

Supplementary Table 3: Deviance table of the effects of experiment and temperature on larval cod survival for Southern Gulf experiments conducted in 2003 and 2011*. *P*-values were obtained from Chi square tests that were used to determine if the model fit improved significantly by sequentially adding population, temperature and their interaction to the null model.

Model term	df	Deviance	Residual df	Residual deviance	*P*	
null			15	683.65	-	
experiment	1	594.49	14	89.16	<0.001	**
temperature	1	5.46	13	83.70	0.328	
experiment × temperature	1	0.04	12	83.66	0.932	

*Survival was measured on day 43 and day 29 in 2003 and 2011, respectively.
Asterisks denote significance at the following levels of α: * = 0.10, ** = 0.05.
