# Supplementary material for: Variation in spawning time promotes genetic variability in population responses to environmental change in a marine fish
Source: Conserv Physiol. 2015 Jul 2;3(1):cov027. doi: 10.1093/conphys/cov027 (PMC4778481; doi:10.1093/conphys/cov027)
Supplement: Supplementary Data [file cov027supp.zip › cov027supp_table4.pdf]

Supplementary Table 4: Mean larval cod lengths at hatch.

Population	Length (mm)	Standard error (mm)
Bonavista	4.41	0.04
Placentia	4.35	0.03
Southern Gulf (2003)	4.52	0.03
Southern Gulf (2011)	4.18	0.03
Fundy	5.05	0.03
Sambro	4.92	0.03
