# Supplementary material for: Variation in spawning time promotes genetic variability in population responses to environmental change in a marine fish
Source: Conserv Physiol. 2015 Jul 2;3(1):cov027. doi: 10.1093/conphys/cov027 (PMC4778481; doi:10.1093/conphys/cov027)
Supplement: Supplementary Data [file cov027supp.zip › cov027supp.pdf]

Supplementary data

Purpose

To (a) determine the number of families represented in the Southern Gulf (2011) and Sambro (2012) common-garden experiments, (b) evaluate whether the number of families varied between the beginning and end of an experiment, and (c) assess whether population reaction norms may be biased due to the presence of large families.

Methods

We genotyped 120 larvae that were sampled on day 0 and all larvae that were sampled for growth measurements at 29 days post hatch. We also collected fin clips from surviving adults after spawning was complete. All samples were stored in 95% ethanol at 4°C and genotyped at five microsatellite DNA loci (Gmo8, Gmo19, Gmo34, Gmo35, and Tch5), following Hardie *et al.* (2006).

We used COLONY v2.0.2.3 (Jones & Wang, 2010) to reconstruct the pedigrees of the spawning adults and larvae from each population. Parental assignment was performed on day 0 and day 29 larval samples combined and the genotypes of candidate parents were supplied, providing COLONY with a larger pedigree from which to determine likelihoods. We used the full-likelihood method with medium precision and a random seed and chose to update the allele frequencies as the analyses progressed to mitigate potential bias due to large families. Each analysis consisted of three simultaneous runs to increase the chances of finding the best configuration with the maximum likelihood and obtain more reliable estimates of uncertainty. We used a per

locus error rate of 0.01 and repeated all analyses using short, long, and very long runs to assess whether the maximum likelihood configuration had been reached.

To assess family-level variability in reaction norm slopes, we performed a two-way ANOVA on a mixed-effects model with temperature and family as fixed effects and tank as a random effect. There is the potential for families present in high proportions in the experiment to bias the resulting reaction norms. To estimate whether the growth reaction norms might be so biased, we performed a two-way ANOVA to compare growth reaction norms for Sambro cod based on two different data sets: 1) all available data, and 2) lengths that had been averaged within families within temperatures (i.e. each family only contributed one mean length value to each temperature treatment).

Results

Microsatellite genotypes were successfully obtained for Sambro day 0 (n=120) and day 29 (n=89) larvae, Southern Gulf day 0 (n=30) larvae and Sambro (n=37) and Southern Gulf (n=4) adults. No Southern Gulf day 29 larvae were genotyped successfully, owing to sample degradation. 278/280 (99%) genotypes were comprised of all five loci and 2/280 (1%) genotypes were comprised of four loci.

Short runs in COLONY produced slightly different results than long runs and very long runs. However, results from the long and very long runs were identical (Southern Gulf) or nearly identical (Sambro – one offspring assignment differed), suggesting that the maximum likelihood configuration had been reached. The results from the very long runs in COLONY are presented here.

Fifteen full-sib families were identified in samples taken from the start of the Southern Gulf experiment, with 1-5 offspring in each family. Nine breeding adults contributed to these families: 4 and 5 members of each sex (Table 1).

Table 1: Number of Southern Gulf larvae assigned to each full-sib family. Row and column totals represent the number of larvae in each half-sib family. Parent IDs are given in the first row and the first column.

	A-1	A-3	*1	*2	Total
A-2	4	0	0	0	4
#1	1	1	5	1	8
#2	1	2	1	1	5
#3	5	2	1	1	9
#4	0	2	2	0	4
Total	11	7	9	3	30

We identified 22 and 20 full-sib families from Sambro day 0 and day 29, respectively. These families were derived from 7 and 9 (day 0; Table 2) and 7 and 6 (day 29; Table 3) breeding adults of each sex. Collectively, 29 full-sib families (from 10 and 9 adults of each sex) were sampled from the Sambro experiment. Family size as a proportion of sample size ranged from 1-49% (mean=5%) for day 0 and 1-36% (mean=4%) for day 29. One family (parents: A-14 and A-39) contributed disproportionately to the Sambro experiment, producing 44% of offspring.

Table 2: Number of Sambro larvae sampled on day 0 assigned to each full-sib family. Row and column totals represent the number of larvae in each half-sib family. Parent IDs are given in the first row and the first column.

	A-14	A-20	A-27	A-29	A-34	A-36	A-41	Total
A-16	0	0	0	0	0	0	1	1
A-23	4	0	0	0	0	0	1	5
A-25	8	1	0	1	1	0	1	12
A-26	7	0	0	0	0	0	0	7
A-35	1	0	0	0	0	0	1	2
A-39	59	5	3	0	0	1	2	70
#1	11	4	2	0	0	0	1	18
#2	0	0	2	0	0	0	0	2
#3	3	0	0	0	0	0	0	3

Total	93	10	7	1	1	1	7	120

Table 3: Number of Sambro larvae sampled on day 29 assigned to each full-sib family. Row and column totals represent the number of larvae in each half-sib family. Parent IDs are given in the first row and the first column.

	A-14	A-15	A-20	A-22	A-27	A-31	A-41	Total
A-16	0	0	0	0	0	0	0	0
A-23	0	0	0	0	0	0	0	0
A-25	14	0	1	0	2	0	0	17
A-26	1	0	1	0	0	0	0	2
A-35	0	0	0	0	0	1	0	1
A-39	32	2	5	1	2	2	3	47
#1	14	0	1	0	2	1	1	19
#2	0	0	0	0	1	0	0	1
#3	2	0	0	0	0	0	0	2
Total	63	2	8	1	7	4	4	89

There was no significant difference in reaction norm slopes among families (F=0.98; *P*=0.46; Table 4). Growth reaction norms for Sambro based on 1) all available data, and 2) lengths that were averaged within families within temperatures were not significantly different from one another (F=0.62; $P_{5,117}$=0.688; Table 5). Therefore, the reaction norm was not significantly biased due to the presence of large families.

Table 4: Effects of family and temperature on larval cod growth for Sambro.

Model term	df	Sum of squares	Mean of squares	F	*P*
family	4	2.26	0.57	1.46	0.19
temperature	1	0.01	0.01	0.01	0.99
family × temperature	4	1.52	0.38	0.98	0.46
**Model term**		**Variance**	**Standard deviation**		
tank		8.21	2.87		
residual		0.40	0.63		

Asterisks denote significance at the following levels of α: * = 0.1, ** = 0.05.

Table 5: Effects of data set and temperature on larval cod growth for Sambro when comparing reaction norms constructed from 1) all available data, and 2) lengths averaged within families within temperatures.

Model term	df	Sum of squares	Mean of squares	F	*P*
data set	1	0.00	0.00	0.00	0.977
temperature	2	1.12	0.56	1.21	0.301
data set × temperature	2	0.30	0.15	0.33	0.722
residuals	117	54.01	0.46	-	-

Asterisks denote significance at the following levels of α: * = 0.1, ** = 0.05.

Summary

At least 15 full-sib families were represented in the Southern Gulf experiment, although the true number of families is likely larger than could be detected by the small sample size that was evaluated. At least 29 full-sib families were represented in the Sambro experiment, with a similar number of families at the beginning and at day 29 of the experiment. One family comprised a large proportion of the Sambro larvae, but its presence did not significantly bias the resulting growth reaction norm.
